# Development and present status of seismic evaluation and seismic retrofit of existing reinforced concrete buildings in Japan

**DOI:** 10.2183/pjab.97.021

**Published:** 2021-07-31

**Authors:** Tsuneo OKADA

**Affiliations:** *1 Professor Emeritus, The University of Tokyo, Tokyo, Japan.

**Keywords:** seismic evaluation, seismic retrofit, existing building, reinforced concrete building, Japan

## Abstract

This paper describes the development and present status of seismic evaluation and seismic retrofit of existing buildings mainly for low-rise and medium-rise reinforced concrete buildings in Japan. First, since the seismic evaluation of existing buildings has close relationships with the seismic design of new buildings, a brief history of the development of seismic design, seismic evaluation, and seismic retrofit is provided in terms of major earthquake disasters mostly in Japan and associated with some major events in the U.S. Then, the development of seismic evaluation and retrofit is reviewed, focusing on the items in which the author has been deeply involved. This provides insight into previous earthquake damage, methodologies for seismic evaluation, the basic concept of the Standard for Seismic Evaluation of Existing Reinforced Concrete Buildings, studies on the demand criteria for seismic safety, and the present status of seismic evaluation and retrofit. Finally, the typical methods of seismic retrofit and some examples of retrofitted buildings in Japan are explained.

## 1 Introduction

Buildings in earthquake-prone areas have been designed and constructed according to seismic codes, including regulations and standards defined in the corresponding countries or regions. Therefore, the seismic performance of existing buildings is usually highly dependent on the seismic codes that were utilized when they were designed and constructed, as the seismic codes are occasionally revised and updated based on the developments in earthquake engineering. Whenever the codes are revised, many existing buildings become outdated and are referred to as “existing nonconforming buildings” that do not satisfy the newly revised seismic codes. However, this does not always mean that all of the nonconforming buildings become vulnerable to earthquakes given the redundancies in their seismic capacities. Therefore, as earthquake countermeasures, it is essential to evaluate the seismic capacities of nonconforming buildings before an earthquake event, to find those with poor redundancies, and to upgrade or reconstruct them. In this paper, finding buildings with seismically poor redundancies through an evaluation of their capacities is referred to as “seismic evaluation”, and upgrading their performance is referred to as “seismic retrofit” or “seismic retrofitting”. The background and history of the development of seismic evaluation and retrofitting, an outline of their procedures, the current status of their applications, *etc.* are described, with a focus on low- and medium-rise reinforced concrete buildings in Japan.

## 2 History of the development of seismic design, seismic evaluation, and seismic retrofitting

In this chapter, a brief history of seismic design for new buildings and seismic evaluation and retrofitting of existing buildings is presented together with major earthquake disasters that mostly occurred in Japan. Major events in the U.S., one of the leading countries in the field of earthquake engineering, are also listed to help international readers understand earthquake countermeasures for buildings in Japan.

**Figure 1.**
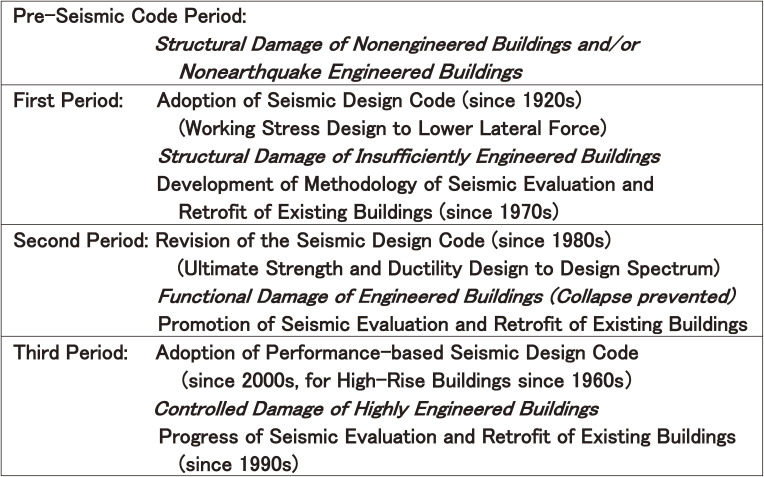
History of earthquake damage, seismic design, seismic evaluation, and seismic retrofit.^1)^

Since seismic codes were developed in earthquake-prone countries at the beginning of the twentieth century, the seismic resistance of buildings has been improved, although their levels vary according to the years of design and construction. To understand how seismic capacity has been improved in approximately the past one hundred years, it may be convenient to divide history into the following four periods: the pre-seismic code period, the first-generation seismic code period (first period), the second-generation seismic code period (second period), and the third-generation seismic code period (third period), according to the development of seismic design, seismic evaluation, and seismic retrofitting methods, as shown in Fig. 1.^1)^

In the pre-seismic code period, most buildings were constructed without any consideration of earthquake engineering criteria, or with some consideration based on the engineers’ own studies and/or experience of past earthquake damage. The buildings in this period are called “nonengineered buildings” or “nonearthquake engineered buildings” and are shown in Fig. 1. Research on earthquake engineering was launched during this period, inspired by serious damage to nonengineered buildings and nonearthquake engineered buildings. This began after the 1891 Nobi earthquake in Japan and the 1906 San Francisco earthquake or the 1925 Santa Barbara earthquake in California, U.S.A.

The first period started in the first half of the twentieth century. Seismic codes were adopted in most earthquake-prone countries or regions beginning in the 1920s. In Japan, the first seismic code was adopted in the Urban Building Law in the year following the 1923 Great Kanto earthquake disaster. In the U.S., the Field Act and Riley Act on seismic design for school buildings and other buildings, respectively, passed the California State Legislature after the Long Beach earthquake occurred in 1933. The seismic design method during this period was based on the so-called working stress design. This method considers an earthquake force to be a static lateral force, calculates the stresses of structural members elastically, and verifies that the calculated stresses are less than the allowable stresses defined according to the strengths of materials in seismic codes. The ratio of static lateral force to building weight is called a seismic coefficient or Sano-seismic coefficient, which was proposed by Sano, T. in 1912.^2)^

In the second half of the twentieth century, some buildings that were designed according to the first-generation seismic codes, labeled as “insufficiently engineered buildings” in Fig. 1, suffered from earthquake damage. For example, building damage due to the 1964 Niigata earthquake and the 1968 Tokachi-oki earthquake in Japan or the 1971 San Fernando earthquake in California, U.S.A. showed that the seismic codes at that time were not sufficient to maintain seismic safety. Therefore, the research and development of earthquake engineering were accelerated, and the revision of seismic design codes began.

The second period started in the 1980s with this code revision. The fundamental concept of the revised seismic design codes was to practically consider the ultimate strength, ductility (deformability), and nonlinear earthquake response of structures. More specifically, the method used a “design seismic force (required strength)” that reduced the elastic earthquake response depending on the ductility of the building. Since the Japanese seismic design code in the Building Standard Law (Urban Building Law before 1950) was revised to upgrade the demand criteria in accordance with this concept in 1981,^3)^ the seismic capacities of buildings have been significantly improved. Similar concepts were adopted in the Mexico City Building Code^4)^ in 1976, the report of the Applied Technology Council (ATC-3-06)^5)^ in 1978, and the Uniform Building Code^6)^ in 1988, among others.

Another important issue that was studied in the last half of the first period and promoted in the second period was the seismic evaluation and retrofitting of existing buildings, which is the main topic of this paper. In Japan, the Standard for Seismic Evaluation and Guidelines for Seismic Retrofit of Existing Reinforced Concrete Buildings^7–9)^ was published in 1977 by the Japan Association for Special Building Safety (JASBS), which has been referred to as the Japan Building Disaster Prevention Association (JBDPA) since 1979, before the 1981 revision of the seismic design code in Japan. Similar standards and guidelines for steel buildings,^10)^ wooden houses,^11)^ and reinforced concrete-encased steel (SRC) buildings^12)^ were published until 1983 by the JBDPA. For government facilities, the Guideline for Seismic Inspection and Repairs of Governmental Facilities in the Comprehensive Earthquake Proof Plan Standardization for Governmental Facilities^13)^ was published by the Building and Repairs Association, which has been referred to as the Public Buildings Association since 1987. However, seismic evaluation and retrofitting were not implemented very often during the second period, except in the limited area of the Shizuoka Prefecture in Japan, until the Great Hanshin-Awaji earthquake disaster, which occurred due to the 1995 Hyogo-ken Nanbu earthquake.

The Great Hanshin-Awaji earthquake disaster triggered the transition to the third period by improving the seismic codes of the second period and further promoting seismic evaluation and retrofitting. In this earthquake, there were very few cases of collapse or serious damage to buildings designed and constructed according to the second-generation seismic codes, but there were several buildings that had to be demolished because they could not maintain their functions after damage; thus, further improvement of the seismic codes was needed. Additionally, since most of the buildings that collapsed or were severely damaged were constructed before the second period, further study and implementation of seismic evaluation and retrofitting were required. To this end, research began to improve the seismic codes for new buildings, and at the same time, the Act on Promotion of Seismic Retrofit of Buildings^14)^ was enacted for existing buildings in 1995. The Seismic Evaluation Standards^7,10–12)^ issued by the JBDPA and the guideline issued by the Building and Repair Association^13)^ were adopted as equivalent methods to the seismic evaluation method defined in the act.

In the U.S., the Greene Act-1 and the Greene Act-2 passed the California State Legislature in 1967 and 1968, respectively, to set a deadline for school districts to inspect the seismic safety of pre-Field Act school buildings. The importance of seismic evaluation and retrofitting of existing buildings was recognized, and studies were carried out. The report of the Applied Technology Council for seismic evaluation of existing buildings (ATC-14)^15)^ was published in 1987. The 1994 Northridge earthquake occurred in California just one year before the 1995 Hyogo-ken Nanbu earthquake in Japan, accelerating seismic evaluation and retrofitting. The report of the Applied Technology Council (ATC-40)^16)^ for the seismic evaluation and retrofitting of concrete buildings and the Handbook for Seismic Evaluation of Buildings (FEMA 310)^17)^ were published in 1996 and 1998, respectively.

The third period started around the end of the last century. The purpose of the third-generation seismic codes was to provide design methods to meet a wide range of seismic performance objectives required for the building process, not just collapse prevention. They aimed to introduce the concept of a performance-based design method, rather than a specification-based design method, into the seismic design.

In Japan, reports to recommend the importance of developing performance-based seismic design codes were issued by the task committee at the Architectural Institute of Japan (AIJ), chaired by the author in 1995^18)^ and 1998,^19)^ and by the Building Council of the Ministry of Construction in 1997.^20)^ In response to the recommendations, the seismic code from the second period would continue to be used with some minor modifications, and a new optional seismic calculation method of estimation of the story displacement response based on the equivalent linear stiffness and damping was added as a performance-based seismic design in the seismic code revised in 2000.^3)^

In the U.S., VISION 2000,^21)^ which focused on a performance-based seismic design, was proposed by the Structural Engineers Association of California in 1995. Several existing model building codes, including seismic codes, were unified into the International Building Code^22)^ in 2000.

In conjunction with these trends, measures for seismic evaluation and retrofitting employed in the second period were updated. In Japan, the Act on Promotion of Seismic Retrofit of Buildings^14)^ was amended in 2005 and 2013, and the Standard for Seismic Evaluation and Guidelines for Seismic Retrofit of Existing Reinforced Concrete Buildings^7)^ was revised in 2007 and 2017. In the U.S., various methods have been integrated; at present, the International Existing Building Code^23)^ and Seismic Evaluation and Retrofit of Existing Buildings (ASCE 41-17)^24)^ are available.

## 3 Lessons from building damage due to the 1968 Tokachi-oki earthquake

**Figure 2.**
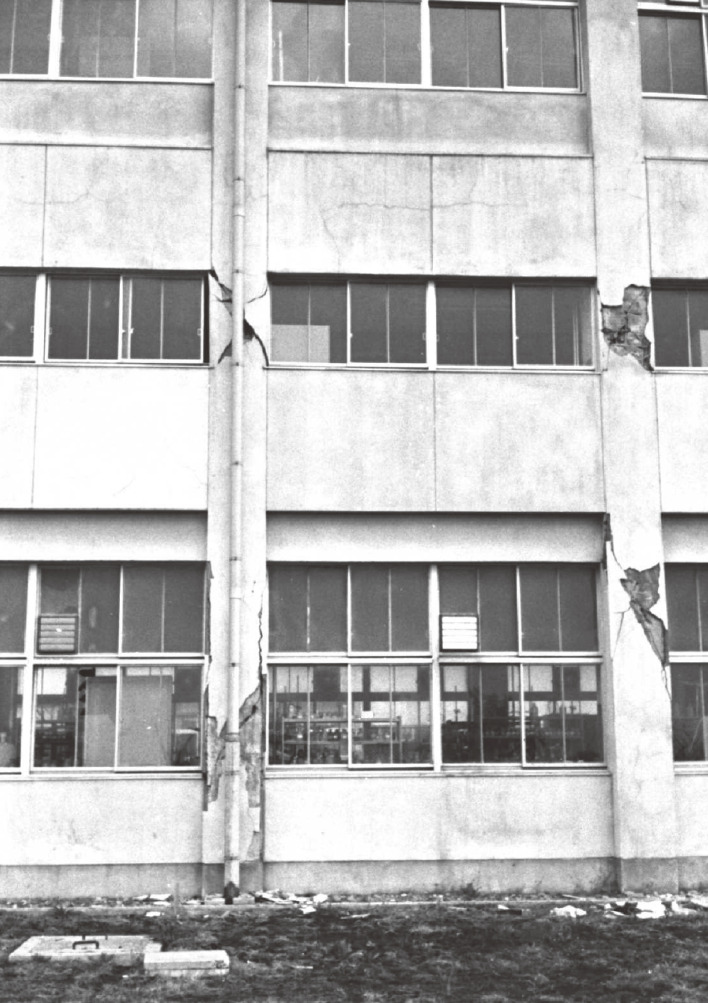
Three-story school building after the 1968 Tokachi-oki earthquake.

As described earlier, bitter lessons from damaging earthquakes have always inspired the earthquake engineering community to develop new concepts and technology and implement them in society. The lessons learned from the buildings damaged by the 1968 Tokachi-oki earthquake accelerated the development of rational methods for seismic design, seismic evaluation, and seismic retrofitting. This chapter provides an overview of the earthquake, the damage, and the lessons learned as a result. The characteristics of the earthquake and damage are as follows^25)^: (1) the hypocenter was located approximately 180 km east of the city of Hachinohe, which was one of the most severely damaged areas, at a depth of 20 km; (2) the magnitude was 6.8 on the Richter scale; (3) the death toll, including those who were missing, was 52; (4) approximately 3,000 wooden houses were severely damaged; (5) approximately 20 newly constructed reinforced concrete buildings experienced severe damage, with a ratio of severe damage to medium damage of approximately 10%; and (6) the maximum ground acceleration observed on the soft ground of the Hachinohe port area was 23% of gravity. Based on these statistics, the disaster was not as serious as recent large-scale earthquakes. However, its impact on professionals in the earthquake engineering community was very strong. Low-rise reinforced concrete school buildings designed according to the existing seismic design codes and constructed with strong workmanship were damaged, as shown in Fig. 2. Most engineers and researchers assumed that such buildings would have had enough seismic resistance due to their redundancy, even though they had been designed to handle static lateral forces equal to 20% of the building weight, which would be smaller than the elastic response of lateral forces due to severe earthquakes.

**Figure 3.**
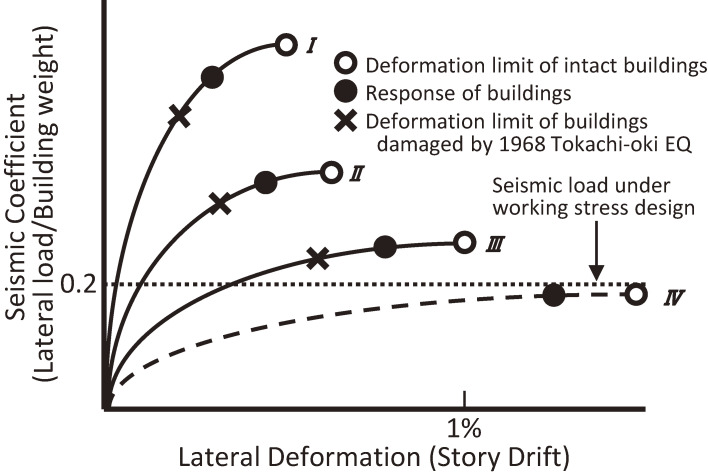
Relationship between the lateral force and lateral deformation of reinforced concrete buildings after an earthquake.^26)^

Investigations to clarify why such damage was caused have been carried out by many researchers and institutions, including AIJ. Figure 3, which simply and accurately expresses these causes, was prepared by the AIJ Task committee^26)^ chaired by Umemura, H. in 1969 and modified by the author. The vertical axis shows the lateral force, representing the earthquake lateral force response in terms of the seismic coefficient, and the horizontal axis shows the lateral deformation in terms of the story drift angle. The curves in the figure schematically show the force–displacement relationships, *i.e.*, the so-called backbone curves, of several types of buildings.

In the figure, reinforced concrete buildings in the damaged area are classified into three types: I, II, and III, showing the characteristics of buildings with many shear walls, with some shear walls, and with less or no shear walls, respectively. Type IV shows the characteristics of high-rise buildings that did not exist in the damaged area. The symbols ○ and × are the deformation limits of intact buildings and damaged buildings, respectively, and the symbol ● shows the earthquake response of the buildings. It is evident that the buildings had various levels of strength, even if they were designed using the same seismic coefficient of 0.2, and that the buildings’ responses varied according to the building type, as shown by the symbol ●. The figure shows that buildings with a deformation limit (ductility), marked by ○, beyond the earthquake response point, marked by ●, survived; otherwise, buildings failed at the point marked by ×. Therefore, the ultimate strength, ductility, and nonlinear earthquake response are recognized as essentials in the future seismic design and have since become important considerations for building engineers.

The Design Guide for High-Rise Buildings^27)^ was published by AIJ in 1964, and the first high-rise building in Japan, the Kasumigaseki Building, was completed in the same year as the 1968 Tokachi-oki earthquake. Thus, researchers and engineers who were interested or engaged in the design of high-rise buildings could have recognized the importance of considering the ultimate strength, ductility, and nonlinear earthquake response in discussing seismic performance at that time.

Therefore, studies analyzing low-rise and medium-rise buildings using nonlinear earthquake response analysis for high-rise buildings were carried out.^28)^ Such an analysis was useful to understand the reasons why buildings were damaged, but it was difficult in practice to use this analysis method for the design of low-rise or medium-rise buildings given the additional complications as compared to high-rise buildings. Therefore, studies to develop more rational but practical design methods that consider the nonlinear dynamic behavior of buildings during earthquakes have been carried out since then.^29–34)^ A consensus on the concept of future seismic design methods was obtained from researchers and engineers around the late 1970s. The main goal was to reduce the seismic force to linear elastic systems according to the ductility of the building and to consider the ductility in determining the ultimate strength required for the building. This idea can be considered a practical application of the results of basic research that has been carried out since the 1960s.^35,36)^

Adopting this concept, the seismic design code was revised in 1981 in Japan,^3)^ where the ratio of the strength required for the building (required strength) to the seismic response force to the linear elastic system, which is usually called a standard seismic force, is defined as the force reduction coefficient (*Ds* coefficient). The same concept was adopted in Mexican and U.S. seismic codes during the second period, where the *Q* factor, *Rw* factor, or *R* factor are employed^4–6)^ to represent the force reduction factor as the reciprocal of the *Ds* coefficient, *i.e.*, the ratio of the standard seismic force to the required strength.

During this period, projects for developing seismic evaluation methods, which will be reviewed in the next chapter, were carried out based on concepts similar to those mentioned above.

## 4 Development of seismic evaluation methods for reinforced concrete buildings

Some of the seismic evaluation methods developed in the 1970s for reinforced concrete buildings are summarized in this chapter.

The method proposed for reinforced concrete low-rise buildings^37)^ by the Building Research Institute estimates the lateral strength of a building from the cross-sectional area of structural elements, such as walls and columns, and judges the seismic safety of the building according to the ductility of the structural elements, which is based on their aspect ratios. The method of estimating the strength is based on Ref. 38, in which the relationships between the cross-sectional areas of walls and columns and damage to buildings from the 1968 Tokachi-oki earthquake were investigated.

Reference 39 is the report prepared by the author and Bresler, B. for the project “Seismic Safety of Existing School Buildings” at the University of California, Berkeley, supported by the National Science Foundation, and for the U.S.–Japan cooperative research program entitled “Earthquake Engineering with Emphasis on the Safety of School Buildings” under the joint sponsorship of the National Science Foundation and Japan Society for the Promotion of Science. A seismic evaluation method for existing reinforced concrete buildings was proposed based on the concept developed for the seismic design of new buildings in Ref. 30, including some examples that are applied to judge the safety of existing school buildings in California, U.S.A. The method of Ref. 30, published in 1973, was used as a reference in the 1981 revision of the Japanese seismic code.

Reference 40 contains four papers written by Bresler, B. *et al.* during an investigation of the methods for evaluating the safety of existing school buildings at the University of California, Berkeley, including the abstract of Ref. 39.

Reference 7 is the Standard for Seismic Evaluation and Guidelines for Seismic Retrofit of Existing Reinforced Concrete Buildings, published by the JASBS (JBDPA since 1979) under the supervision and sponsorship of the Ministry of Construction, which has been referred to as the Ministry of Land, Infrastructure, Transport and Tourism since 2001; the subject of this reference is one of the main topics of this paper. To develop the standard and the guidelines, an advisory committee chaired by Umemura, H. and two subcommittees, one to draft the standard chaired by the author and the other to draft the guidelines chaired by Hirosawa, M., were established in the JASBS. The seismic evaluation method adopted in the standard analyzes the seismic performance of existing reinforced concrete buildings using the *Is*-index, the concept of which will be described briefly in the next chapter. The standards and guidelines have been revised three times, in 1990, 2001, and 2017, and even now, they are used for most seismic evaluation and retrofitting projects in Japan.

As mentioned in the Introduction, seismic evaluation is applied to evaluate the redundancy of buildings designed with older seismic codes. In general terms, it is one way for seismic evaluation to confirm the safety of buildings designed under the first-generation seismic code by redesigning them with the second-generation seismic code. However, this method has the following drawbacks: (1) seismic codes usually include uncalculated specifications, and buildings designed according to older codes are often judged unsafe because they do not meet the current specifications; (2) redesigning existing buildings according to updated seismic codes is not practical because it is more complicated and time-consuming than a new construction; and (3) the deterioration of seismic performance over time is not taken into account. Seismic evaluations that do not require seismic redesigns are therefore needed. Additionally, the Seismic Evaluation Standard preemptively employed the concept of the second-generation seismic code. Therefore, it was acceptable for practical application, although it was quantitatively less accurate due to its simplified procedure. These are the reasons why the Seismic Evaluation Standard formulated in the first period is still in use today, in the third period, with repeated revisions.

## 5 Main ideas of the Standard for Seismic Evaluation of Existing Reinforced Concrete Buildings (1977, 1990, 2001, and 2017)

The Standard for Seismic Evaluation of Existing Reinforced Concrete Buildings (Seismic Evaluation Standard)^7)^ consists of three different levels of procedures: first-level, second-level, and third-level procedures. The first-level procedure is the most simplified while the third-level procedure requires the most elaborate calculations. These three evaluation procedures share the same basic concept, which is described in the following section, and the second-level procedure has been most widely applied to existing buildings in Japan to date.

In the standard, the seismic performance of a building is expressed by the seismic index of the structure (*Is*-index) for each story and each direction, as will be described next, and better performance is expected for a higher index value. The story of a building with an index higher than the seismic demand index (*Iso*-index) is deemed safe. The definition of the *Iso*-index will be discussed in Chapter 6.

The *Is*-index represents the seismic force level that a building can survive and is defined by the product of three indices: a basic seismic index of structure (*E*_0_-index), which is calculated from the lateral strength and ductility (or deformability); an irregularity index (*S*_*D*_-index), which accounts for disadvantages due to irregularities in the plane and along the height of a building; and a time index (*T*-index), which considers the deterioration of strength and ductility after construction.

$$Is=E_{0}\times S_{D}\times T.$$ [1]

The *E*_0_-index is given by the product of the strength index (*C*-index) and the ductility index (*F*-index), which denote the lateral strength and deformability of a building, respectively. The *E*_0_-index for structures having members with different deformability values (*e.g.*, flexible columns and stiff walls) is calculated based on the *C*-index and *F*-index of each member in the story.^8,41)^ The *F*-index is formulated^42)^ based on a similar concept to the 1/*Ds* in the Japanese seismic code and the *Q*, *Rw*, or *R* in the Mexican and U.S. codes. A checklist is provided to find other indices, the *S*_*D*_-index and *T*-index, to eliminate complicated calculations.

**Figure 4.**
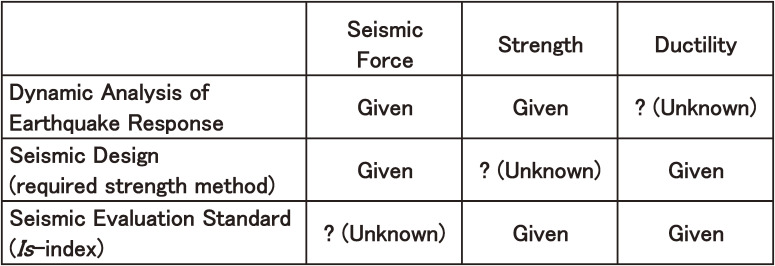
Comparison of the dynamic analysis of the earthquake response, seismic design based on the required strength method and seismic evaluation.^43)^

This is a simplified method to predict the nonlinear earthquake response of a building without performing dynamic response computation, although the basic concept of estimation of the *Is*-index is the same as those of the dynamic analysis of the earthquake response for high-rise buildings and the seismic design based on a required strength method in the second period. However, this approach has a distinctive feature in the adoption of the unknown factor or solution, as shown in Fig. 4.^43)^ As discussed in Chapter 3, seismic force, strength, and ductility are the three major parameters that govern the nonlinear earthquake response of a building. Figure 4 shows the relationship between parameters that are/are not given in the dynamic analysis of earthquake response, the seismic design based on the required strength method, and the Seismic Evaluation Standard.

In the dynamic analysis of the earthquake response, shown in the top row of Fig. 4, the seismic force is given as the expected earthquake ground motion, the strength is given as a nonlinear restoring force parameter, and the solution or unknown obtained by solving a nonlinear vibration equation is usually the story drift. If the drift is smaller than the deformation limit (ductility), the building is deemed safe for the ground motion. In the seismic design based on the required strength method, shown in the middle row, the seismic force is given as a standard seismic force, the ductility is considered in the force reduction coefficient or force reduction factor, and the solution is the required strength for the building. In the Seismic Evaluation Standard in the bottom row, the strength is given as the *C*-index, the ductility is considered in the *F*-index, and the solution is the *Is*-index, showing the level of the seismic force corresponding to the standard seismic force in the required strength method when a story drift reaches a deformation limit of the building. Namely, since the *F*-index of the wall that fails under shear force is defined as 1.0, the *Is*-index can be considered equivalent to the nonlinear seismic response force when the response displacement of the shear wall reaches the assumed deformation limit of 0.4% in terms of the story drift angle, as described in the commentary of the standard.

It might be obvious that the dynamic analysis and the seismic design methods in the second period cannot start before determination of the seismic force obtained from the intensity and characteristics of the earthquake ground motion. However, the *Is*-index in the Seismic Evaluation Standard can be calculated without determination of the intensity and characteristics of the earthquake ground motion because the solution itself presents these results. It is possible to rank buildings and/or stories in terms of seismic safety without predetermining the expected ground motion.

When the evaluation standard was developed, discussion about the intensity and characteristics of expected earthquake ground motions continued, and a consensus had not yet been reached in Japan. Therefore, it was considered that a proposal to estimate the relative seismic performance would be more acceptable to stakeholders such as engineers, building owners, and authorities than to judge a structure as seismically safe or unsafe, as seismic safety depends on the intensity of the ground motions and the level of acceptable damage. Furthermore, since this was a newly developed method, it was necessary to apply it to damaged buildings to verify its accuracy. For those reasons, the value of the seismic demand index (*Iso*-index) was not determined at this time but was left for future study. However, several examples of *Is*-indices of buildings damaged by the 1968 Tokachi-oki earthquake were estimated and are included in the appendix of the Seismic Evaluation Standard^7)^ as a reference for stakeholders.

## 6 Studies on the criteria for judging the seismic safety of reinforced concrete buildings for the Seismic Evaluation Standard

As mentioned above, the first edition of the Seismic Evaluation Standard did not specify the value of the *Iso*-index, but it was formally adopted in the 1990 revision based on the results of various studies. In this chapter, several studies on the criteria for judging the seismic safety of existing reinforced concrete buildings based on the *Is*-index are reviewed.

### 6.1 *Is*-indices of buildings after the 1968 Tokachi-oki earthquake.

**Figure 5.**
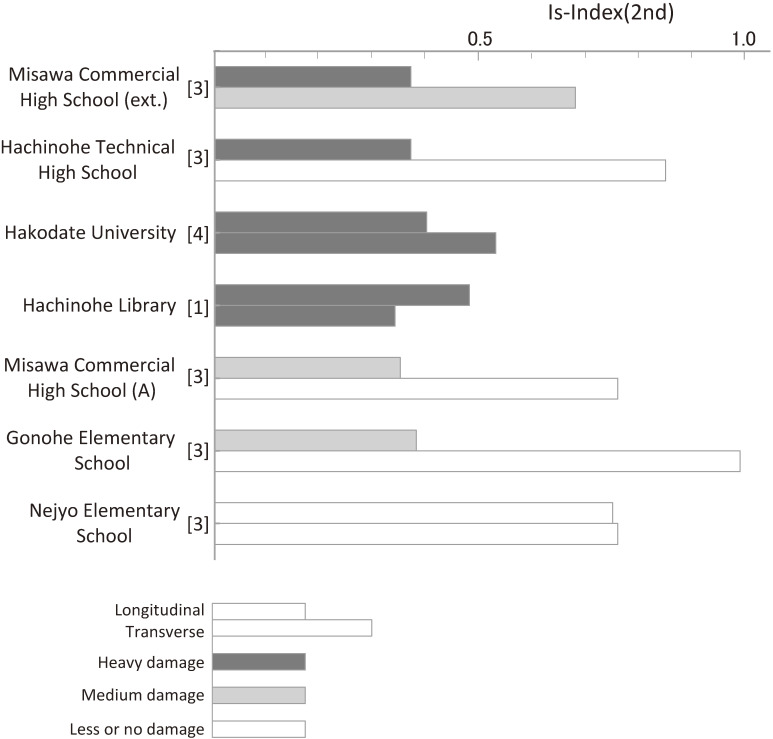
Relationships between the *Is*-indices and building damage due to the 1968 Tokachi-oki earthquake.^7)^ (Numerals in square brackets denote the numbers of stories.)

Figure 5 shows the *Is*-indices obtained through the second-level procedure for seven buildings compiled in the appendix of the Seismic Evaluation Standard^7)^ to investigate the relationships between the *Is*-indices and building damage. The black bars show heavy damage, shaded bars show medium damage, and white bars show little or no damage. This figure suggests that an *Is*-index of more than 0.7 might be recommended to prevent medium or more damage from the level of ground motion sustained during the 1968 Tokachi-oki earthquake. However, as mentioned previously, the judging criterion was not determined in the first edition of the Standard due to a lack of sufficient application data. As mentioned in Chapter 3, the maximum observed ground acceleration was 23% of gravity; however, nonlinear earthquake response analyses of heavily damaged buildings suggested that the ground motions at the bases of those buildings would be larger than those recorded because of differences in the soil conditions.^44,45)^

### 6.2 Seismic safety index (*E_T_*) in the Shizuoka Prefecture.

The Shizuoka Prefecture launched the earthquake countermeasure project for a hypothetical earthquake called the Tokai earthquake in 1976. A seismologist issued a warning,^46)^ and a group of seismologists supported the high possibility of the occurrence of a large earthquake in the near future. The magnitude could be approximately 8 on the Richter scale, and the hypocenter would be just beneath the Shizuoka Prefecture, located near the north end of the Philippine Sea plate and the Nankai Trough. One of the important measures immediately taken after the warning was the seismic evaluation and retrofitting of existing buildings.

To support the Tokai earthquake countermeasures in the Shizuoka Prefecture, the advisory committee chaired by Umemura, H., with the author as the secretary general, called the Committee for Seismic Performance of Reinforced Concrete Buildings, was established in the JASBS in 1977. One of the projects carried out by the committee was to draft the criteria for judging seismic safety of reinforced concrete buildings in the Shizuoka Prefecture. The task committee chaired by Murakami, M. was established and proposed the seismic safety index (*E*_*T*_)^47,48)^ for the seismic demand index (*Iso*-index) in the Shizuoka Prefecture. Simplifying the proposed criteria, the Shizuoka Prefecture issued the guideline in 1979.^49)^

The proposed seismic safety index (*E*_*T*_) is estimated using Eq. [2].

$$E_{T}=Es\times Co\times C_{I}.$$ [2]

The *Es*-index is a basic seismic safety index and is defined depending on the number of stories, the predominant period of ground in seconds, and the failure type (shear failure/flexural failure) of a building, as shown in Table 1. Values in Table 1 show the *Es*-index for buildings failing under shear, while those in parentheses are for buildings failing under flexure. They were obtained using data in Ref. 50, where numerical simulations were parametrically performed on the relationship between the ground motion and the strength required to keep the nonlinear response of a structure lower than a certain acceptable displacement. These values are the criteria to avoid medium or higher damage under the ground motion levels assumed in the Hachinohe city during the 1968 Tokachi-oki earthquake or in the Sendai city during the 1978 Miyagi-ken-oki earthquake. Note that 1.5 times these values are required in the areas closest to the epicenter of the Tokai earthquake. *Co* and *C*_*I*_ are the modification factors that allow for the amplification of ground motion due to site configuration, ranging from 1.0 to 1.25, and the importance factors ranging from 1.0 to 1.5, respectively.

### 6.3 Application of the Seismic Evaluation Standard to reinforced concrete buildings suffering from earthquake damage.

In 1978, several earthquakes hit Japan, and many buildings were damaged. One earthquake was the Izu-Oshima Kinkai earthquake in the Shizuoka Prefecture on January 14, which was not a scenario under the Tokai earthquake study, and the other was the Miyagi-ken-oki earthquake on June 12. Studies applying the Seismic Evaluation Standard to reinforced concrete buildings suffering from earthquake damage were carried out.^51,52)^

**Figure 6.**
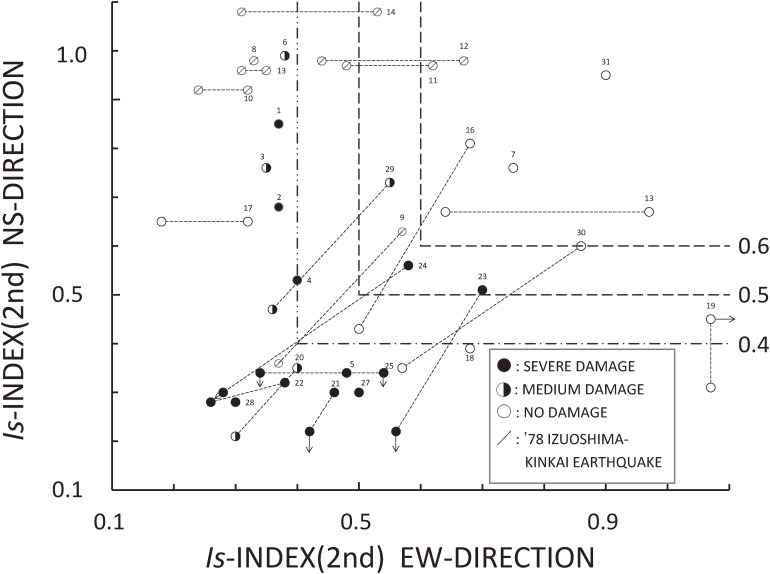
*Is*-indices in the second-level procedure *vs.* earthquake damage of reinforced concrete buildings suffering from the 1968 Tokachi-oki earthquake, 1978 Izu-Oshima Kinaki earthquake, and 1978 Miyagi-ken-oki earthquake.^9,53)^

Figure 6 shows the *Is*-indices from the second-level screening procedure of buildings in several cities along the shore of the Izu Peninsula in the Shizuoka Prefecture and in the Sendai city in the Miyagi Prefecture, which suffered from the earthquakes mentioned above, along with those of buildings that suffered from the 1968 Tokachi-oki earthquake.^9,53)^ The black circle (●), half-black circle (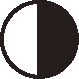), and white circle (○) correspond to severe damage, medium damage, and little or no damage, respectively. From the figure, it is suggested that *Is*-indices of 0.5 to 0.6 in the second-level screening procedure represent the difference between damaged and undamaged buildings that experienced ground motions of approximately 30% of gravity. However, one problem is a lack of data for undamaged buildings.

### 6.4 Studies on the judging criteria of the *Is*-index based on the reliability theory.

As described in section 6.2, the seismic evaluation of reinforced concrete buildings in the Shizuoka Prefecture started in 1977, just after the Seismic Evaluation Standard was published. To help accelerate the use of this standard, a computer program to calculate the *Is*-index of the first-level and second-level screening procedures was developed in 1978 by the task committee chaired by the author in the Committee for Seismic Performance of Reinforced Concrete Buildings. The program was released by the JBDPA under the sponsorship of the Shizuoka Prefecture in 1980.^54)^ Utilizing this program, the *Is*-indices of many public buildings in the Shizuoka Prefecture were estimated by local structural engineers. Studies comparing and combining the *Is*-index data of buildings in the Shizuoka Prefecture and those of damaged buildings in past earthquakes were carried out.^55–57)^

**Figure 7.**
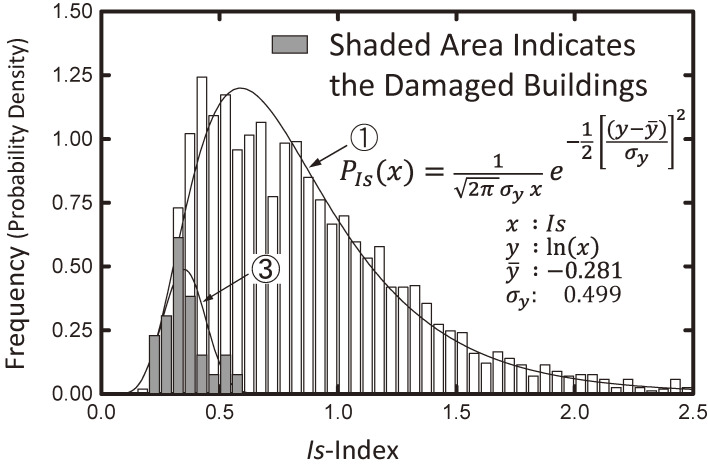
Distribution of the *Is*-indices for existing and earthquake-damaged buildings.^56,57)^

Figure 7 shows the distribution of *Is*-indices of existing buildings cited from Refs. 56 and 57. The bar chart in Fig. 7 represents the frequency of *Is*-indices on the first story based on the second-level screening procedure of approximately 1,600 public buildings, mostly reinforced concrete three- or four-story school buildings, in the Shizuoka Prefecture. The number of *Is*-indices is approximately double the number of buildings because the *Is*-indices are calculated in both the longitudinal and transverse directions of the floor plan. Curve ① is the probability density function approximating the bar charts using a log-normal distribution function. The shaded bar chart in Fig. 7 is the frequency of the *Is*-indices of buildings suffering from medium and severe damage described in section 6.3. The total number of damaged building data points is adjusted to 10% of the number of building data points in the Shizuoka Prefecture, as the damage ratios in past earthquakes are estimated to be approximately 10%. Figure 7 suggests that a seismic safety index (*E*_*T*_), which is the same as the *Iso*-index of the Seismic Evaluation Standard, of approximately 0.6–0.7 is the lower bound to prevent medium or severe structural damage from the seismic intensity levels of past earthquakes, such as the 1968 Tokachi-oki and/or 1978 Miyagi-ken-oki earthquakes, as shown in the previous sections.

**Figure 8.**
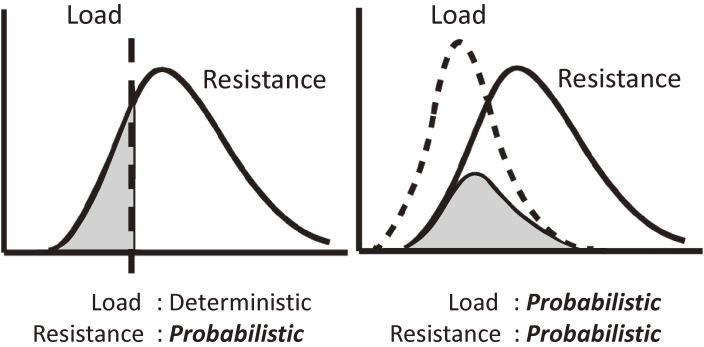
Expected distributions of the resistance (*Is*-indices) of damaged buildings when the load (*E*_*T*_-indices) is deterministic or probabilistic.^56,57)^

**Figure 9.**
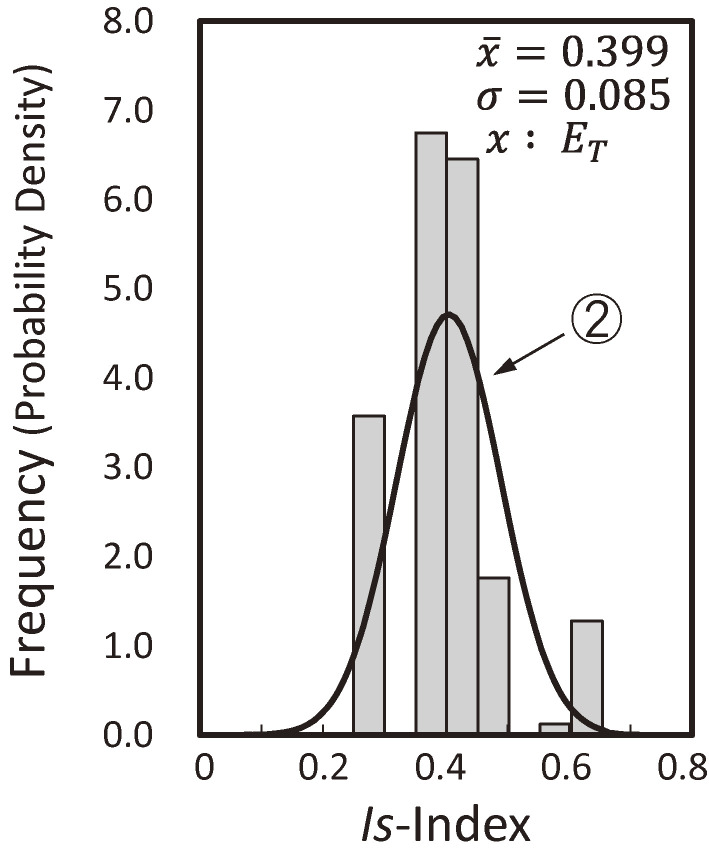
Expected distribution of *E*_*T*_ indices.^56,57)^

Figure 7 also suggests that the seismic safety index (*E*_*T*_) may not be considered deterministic but probabilistic, as illustrated in Fig. 8. Therefore, the distribution of the *E*_*T*_ indices is estimated using the reliability theory according to curve ① and the shaded bar chart of the adjusted distribution of the *Is*-indices of damaged buildings in Fig. 7 and shown by the shaded bar chart in Fig. 9, cited from Refs. 56 and 57. The probability density function approximated by a normal distribution function is given by curve ② in Fig. 9.

**Figure 10.**
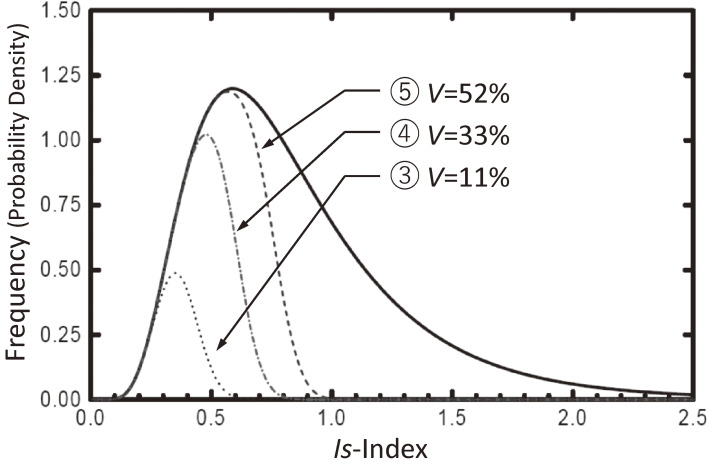
Estimated damage ratio *V*.^56,57)^

Curve ③ in Fig. 7 is the estimated distribution of the *Is*-indices of the damaged buildings calculated by curve ① in this figure and curve ② in Fig. 9, where the estimated damage ratio becomes 11% instead of 10%. Assuming that the *E*_*T*_ index is proportional to the level of ground motion, the mean value of curve ② is increased by approximately 1.5 times and 2.0 times, keeping the variance values constant, and the distribution of buildings that are expected to experience damage is calculated again by the reliability theory, as shown in Fig. 10 and cited from Refs. 56 and 57. Curve ③ is a duplicate of curve ③ in Fig. 7, and curves ④ and ⑤ are the expected distributions of the damaged buildings when the level of ground motion reaches approximately 1.5 times and 2.0 times that in past earthquakes, respectively. The damage ratios are not proportional to the level of the ground motions but instead increase by 3 times and 5 times, respectively. This tendency corresponds to the experience obtained from past earthquake disasters.

The methodology mentioned above has been used in the damage assessment of the Tokai earthquake in the Shizuoka Prefecture since 1993.^58)^

### 6.5 Adoption of the seismic demand index (Iso).

In view of the studies mentioned above, a seismic demand index (*Iso*) was adopted in the Seismic Evaluation Standard^7)^ in 1990. The level of this seismic criteria may be deemed approximately on the same level of the seismic code of Japan in the second period. The seismic safety of a building is judged as follows:

When Eq. [3] is satisfied, the building and/or the story may be judged as seismically safe.

Is≧Iso [3]

where *Iso* = *Es* · *Z* · *G* · *U*, *Es*: 0.8 for the first-level screening, *Es*: 0.6 for the second-level screening, *Es*: 0.6 for the third-level screening, *Z*: zone index (same values as employed in the seismic code), *G*: ground index (amplification factor to allow for the site configuration), and *U*: usage index (*i.e.*, importance factor).

## 7 Building damage due to the 1995 Hyogo-ken Nanbu earthquake and the actions taken after the earthquake

Even in the second period, buildings were damaged due to earthquakes such as the 1983 Nihonkai-Chubu, 1987 Chiba-ken Toho-oki, and 1993 Hokkaido Nansei-oki earthquakes in Japan. Most of the seriously damaged buildings were constructed in the first period or in the pre-seismic code period, as expected. These buildings would have been evaluated as vulnerable if their seismic capacity had been evaluated before the earthquakes. However, since the Building Standard Law of Japan, where the seismic code is involved, is not retroactive, the seismic evaluation and retrofitting of existing buildings have not spread on a country-wide basis, except for the Shizuoka Prefecture. Therefore, many vulnerable buildings were left without seismic evaluation and/or retrofitting until the 1995 Hyogo-ken Nanbu earthquake.

The disaster due to the Hyogo-ken Nanbu earthquake, called the Great Hanshin-Awaji earthquake disaster, brought a drastic change to the processes of seismic evaluation and retrofitting. Awareness has spread nationwide on the importance of seismic evaluation and seismic retrofitting for earthquake disaster mitigation, which has been accelerated since then. As mentioned in Chapter 2, it was reported that most of the buildings that were severely damaged or collapsed were those designed before the second period, and most of the deaths out of more than 6,400 were caused by damaged buildings or houses.^59,60)^ The vulnerability of existing buildings was also verified by studies applying the Seismic Evaluation Standard to buildings suffering from earthquake damage, which is described in Chapter 8. Recognizing the serious vulnerability of older buildings, the Government of Japan immediately enacted the Act on Promotion of Seismic Retrofit of Buildings in December 1995, which is described in Chapter 9.

## 8 Seismic performance of reinforced concrete school buildings in the 1995 Hyogo-ken Nanbu earthquake

**Figure 11.**
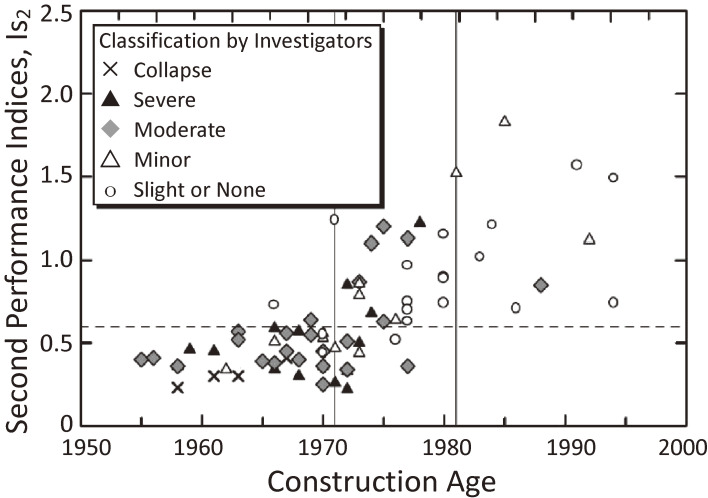
*Is*-indices (second) *vs.* the construction years of reinforced concrete school buildings with damage grades due to the 1995 Hyogo-ken Nanbu earthquake.^61,62)^

Some studies to verify the relationship between the damage grades and *Is*-indices of reinforced concrete school buildings damaged in the 1995 Hyogo-ken Nanbu earthquake are explained here. Table 2 shows the damage statistics of reinforced concrete school buildings in the disaster area as investigated by the task committee of the AIJ, chaired by the author.^61,62)^ Among 631 reinforced concrete school buildings, 8% experienced severe damage or collapse, and 30% suffered from moderate damage or higher. Since a minor revision was made in the seismic code in 1971 before the major revision in 1981, this table is divided into pre-1971, 1971–1981, and post-1981 sections. No serious damage occurred to the school buildings constructed in the second period after 1981. The *Is*-indices of approximately 100 randomly selected school buildings were calculated, and the relationships between the *Is*-indices and the damage grades were investigated, as shown in Fig. 11. The *Is*-indices indicated that the seismic capacities increased for more recent construction years and that the *Is*-indices of most heavily damaged buildings were less than 0.6.

**Figure 12.**
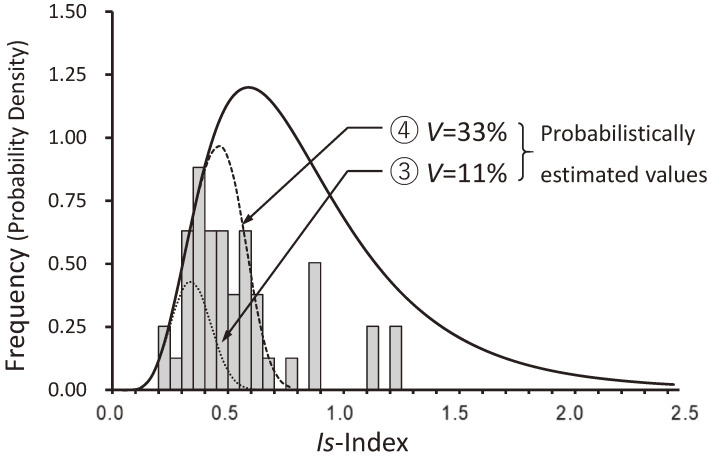
Distribution of the *Is*-indices of damaged reinforced concrete buildings. (Comparison between the estimates and observations for the 1995 Hyogo-ken Nanbu earthquake.^63)^)

Figure 12^63)^ shows the distribution of *Is*-indices of buildings that suffered from moderate damage or higher, adjusting the damage ratio to 29% on curve ① in Fig. 7. The damage ratio in Table 2 was 30%, but it was corrected to 29% by a later investigation. According to Figs. 11 and 12, it should be pointed out that (1) an *Is*-index of 0.6 is the border between severe damage and moderate damage, (2) an *Is*-index of approximately 0.9 is needed to prevent moderate damage, and (3) the intensity of ground motions might be approximately 1.5 times those of the 1968 Tokachi-oki earthquake and the 1978 Miyagi-ken-oki earthquake. This is because the distribution of damaged buildings could be approximated by curve ④ in Fig. 10, obtained by assuming that the level of ground motion is approximately 1.5 times that of curve ② in Fig. 9.

From these studies, it is strongly suggested that the damage to reinforced concrete school buildings could have been significantly mitigated if their seismic capacities had been evaluated and seismic retrofitting had been conducted on the buildings judged unsafe before the earthquake occurred. Unfortunately, no school buildings had been seismically evaluated in the area affected by the disaster.

## 9 Act on Promotion of Seismic Retrofit of Buildings^14)^ and the development of seismic evaluation and retrofitting

The Act on Promotion of Seismic Retrofit of Buildings, enacted in 1995, required that seismic evaluation be conducted by the owners of buildings constructed before 1981 and having a large size, such as more than two stories and more than 1,000 m^2^ of floor area, and a large number of occupants such as schools, gymnasiums, hospitals, theaters, stadiums, auditoriums, department houses, office buildings, *etc.* The act was amended twice, in 2006 and 2013. The latest act required seismic evaluation by owners of all existing buildings, including single-family houses. The act also set the deadline to implement seismic evaluation by the owners of buildings classified as the highest priority such as hospitals, shops, hotels, schools, nursing homes for the aged, *etc.* The *Is*-indices of such buildings and the retrofitting or demolishing schedules of the buildings judged vulnerable are disclosed periodically by local governments.

Since the act was enacted, seismic evaluation and seismic retrofitting have been promoted rapidly. For example, approximately eighty-five thousand public school buildings were constructed before 1981, approximately 65% of which still existed in 2004.^64)^ However, the Ministry of Education, Culture, Sport, Science and Technology recently reported on their website that almost all of them have been evaluated as seismically safe, including retrofitted or reconstructed buildings (https://www.mext.go.jp/content/20202803-mxt_sisetujo-000009172_01.pdf, in Japanese).

However, the seismic evaluation of private buildings has progressed more slowly than that of public buildings. A 2018 report on the website of the Ministry of Land, Infrastructure, Transport and Tourism estimates that approximately fifty thousand buildings accommodating a large number of people and constructed in or before the first period were left vulnerable (https://www.mlit.go.jp/jutakukentiku/house/jutakukentiku_house_fr_000043.html, in Japanese).

## 10 Examples of seismic retrofitting

**Figure 13.**
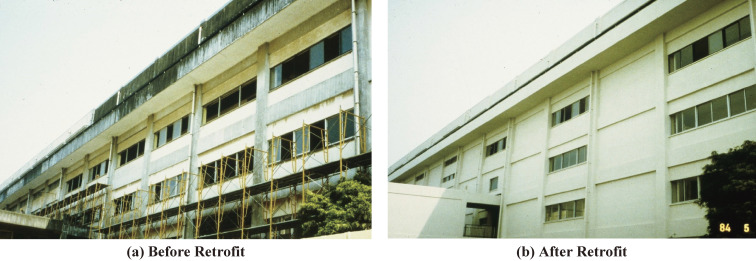
Reinforced concrete school building strengthened by shear walls. (Courtesy of Dr. Masaya Murakami) (a) Before retrofit and (b) after retrofit.

**Figure 14.**
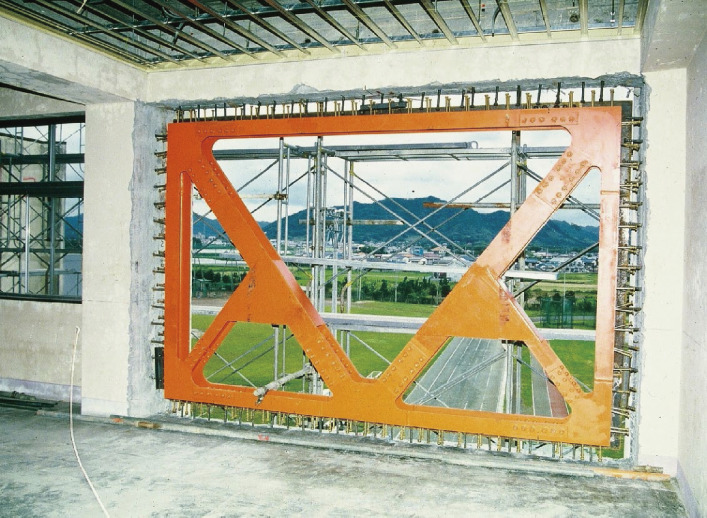
Reinforced concrete school building strengthened by a steel-braced frame. (Under construction; cement mortar is filled between the existing reinforced concrete frame and the steel-braced frame.)

**Figure 15.**
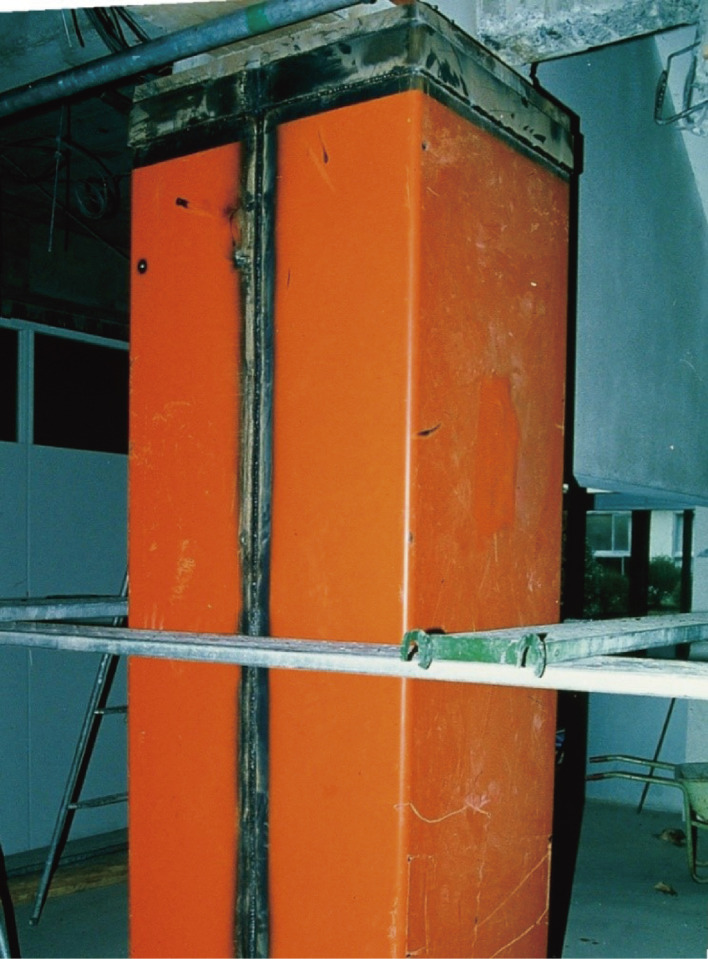
Reinforced concrete column retrofitted by steel plate jacketing to increase ductility.

Buildings judged vulnerable by seismic evaluation should be strengthened, demolished, or reconstructed. Various methods to retrofit reinforced concrete buildings have been developed. Typical methods recommended in the Guidelines for Seismic Retrofit are (1) installing reinforced concrete walls to increase the strength, (2) installing steel-framed braces to increase the strength, and (3) jacketing columns with steel plates or carbon fiber sheets to increase the ductility, as shown in Figs. 13, 14, and 15, respectively. To connect a new structural element to an existing structure, post-installed anchor bolts are usually used, as shown in Fig. 14. Various methods based on concepts similar to those mentioned above have been developed, including patented methods.

Although there are few data to quantify the effectiveness of such seismic retrofitting, most of the buildings that collapsed or were severely damaged in the 2004 Niigata-ken Chuetsu earthquake, the 2011 Great East Japan earthquake, or the 2016 Kumamoto earthquake were vulnerable buildings designed under codes from before the second period, and few of the damaged buildings had been retrofitted before the events. As mentioned earlier, Shizuoka Prefectural Government has been taking measures for seismic evaluation and retrofitting for a long time in anticipation of the Tokai earthquake, and seismic countermeasures have been completed for almost all public buildings. Fortunately, the Tokai earthquake has not yet occurred. However, a series of countermeasures taken by the Shizuoka Prefectural Government has become a role model for other local governments.

Recently, a seismic base isolation system has been utilized for retrofitting, primarily to preserve the original architectural design and interior. To reduce the seismic response of the building, seismic isolators with supplementary vibration damping devices are installed between the existing building and a newly constructed foundation on the ground. For seismic isolators, laminated rubber bearings composed of alternate layers of rubber and steel plates, friction bearings, or ball bearings are usually used. Lead, steel, or oil dampers are also used to increase damping. In such cases, the Seismic Evaluation Standard is applied when seismically evaluating the original building, but advanced methods such as time-history response analysis are used when confirming the seismic performance of the retrofitted building. Examples for retrofitting with base isolation systems can be found in the following projects, where the author was involved as an advisor.

**Figure 16.**
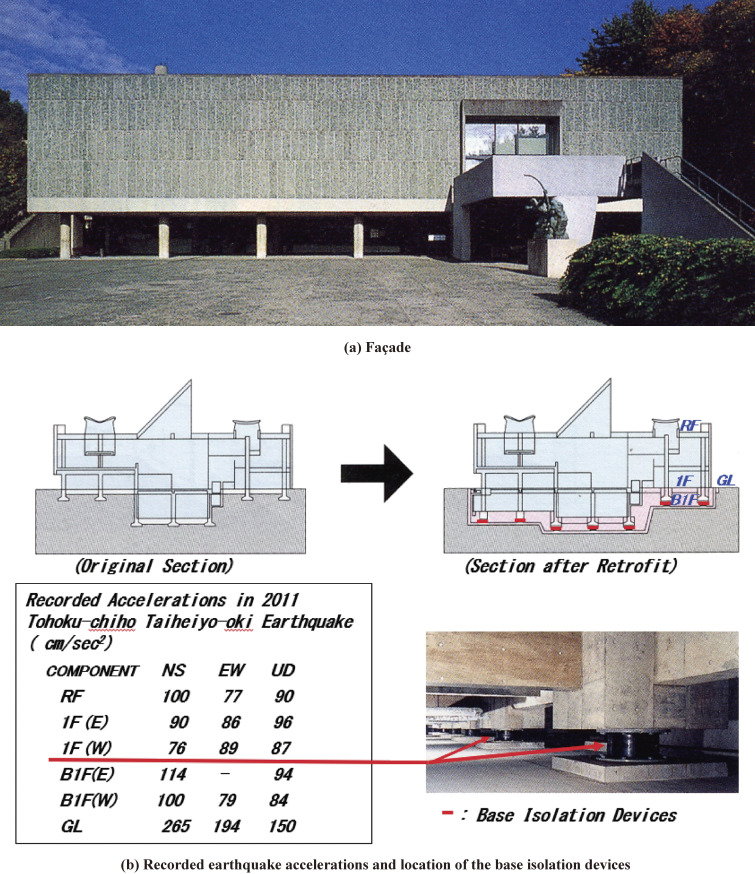
Main building of the National Museum of Western Arts. (Courtesy of the Government Buildings Department, Kanto Regional Development Bureau, Ministry of Land, Infrastructure, Transport and Tourism.) (a) Façade^65)^ and (b) recorded earthquake accelerations^69)^ and location of the base isolation devices.^65)^

The National Museum of Western Art is a reinforced concrete three-story building with a basement constructed according to the design of the world-famous architect Le Corbusier in Tokyo in 1959, as shown in Fig. 16(a). Since it was designed and constructed according to the first-generation seismic code, the level of the *Is*-index did not satisfy the demand criteria. Therefore, the museum building was retrofitted in 1998 with a base isolation system using high damping laminated rubber bearings to preserve the original architecture.^65–68)^ This was the first trial of the base isolation system for seismic retrofitting in Japan. Thus, strong-motion seismometers have been installed by the Building Research Institute, and the accelerograms due to the 2011 Great East Japan earthquake disaster were recorded.^69)^ As shown in Fig. 16(b), the accelerations measured approximately 250 cm/sec^2^ on the ground surface near the building and approximately 100 cm/sec^2^ at the newly constructed basement, which supported the base isolation devices, at the first floor, and at the top floor. In other words, the seismic motion applied to the building base was not amplified even at the top of the building, where it would be expected to be double or triple if the base isolation system had not been installed. The building had no damage and was registered as a world heritage site in 2016. This is a rare example where the impact of seismic retrofitting has been verified quantitatively.

**Figure 17.**
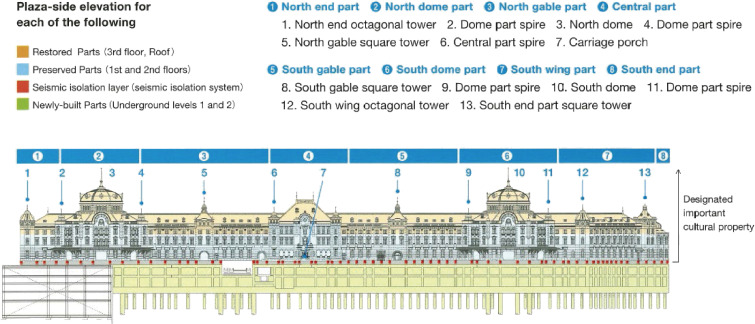
Base isolation of the Tokyo Station Marunouchi Building.^70)^ (Courtesy of the East Japan Railway Company.)

The second example is the Tokyo Railway Station Marunouchi building constructed in 1914 according to the design of Tatsuno, K., who was one of the pioneers of modern architecture in Japan. The original building was a three-story building with two domes and a partial basement made of a brick-infilled steel structure. However, the top floor and domes were destroyed by bombing in 1945; the building was repaired as a two-story building without domes in 1947. Based on the historic and cultural value, it was decided to restore the building to its original design in 2002. The project to restore and retrofit it using a base isolation system was completed in 2012, as shown in Fig. 17. A two-story reinforced concrete-encased steel (SRC) underground structure was constructed to support the base isolation devices. The building is 335 meters long and approximately 20 meters wide, and so 352 isolators and 158 oil dampers were installed. Retrofitting work was performed while maintaining the functions of the railway station during construction.^70,71)^

**Figure 18.**
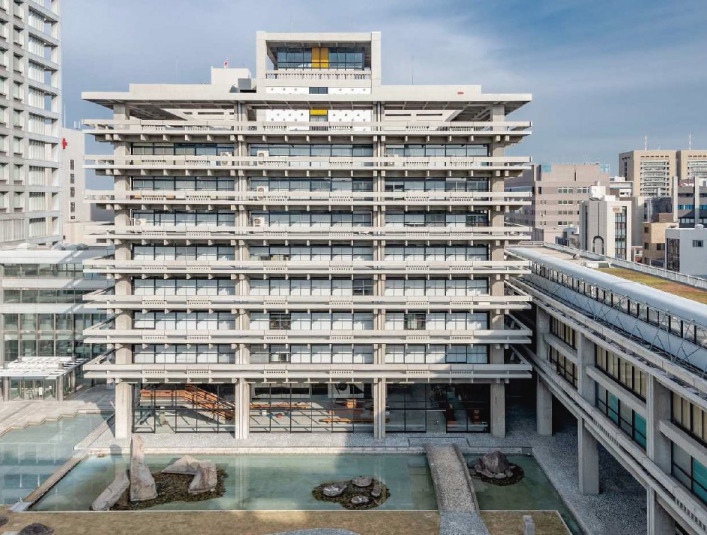
Kagawa Prefectural Office east building. (Courtesy of Kagawa Prefectural Government.)

The last example is the east building of the Kagawa Prefectural Office in the Takamatsu city designed by Tange, K. in collaboration with Tsuboi, Y. for structural design and constructed in 1958. Since this building is one of the distinguished examples of modernist architecture in Japan,^72)^ a base isolation system was used for retrofitting in 2019 to preserve the original architecture^73–75)^ as shown in Fig. 18.

## 11 Concluding remarks

Nearly half a century has passed since the author started studying the seismic evaluation and retrofitting of existing reinforced concrete buildings. As described in this paper, the author has been developing methods for seismic evaluation and retrofitting, verifying them, and disseminating them through JBDPA. Unfortunately, however, during the first two decades, seismic evaluation was not a matter of public concern except in limited areas, such as the Shizuoka Prefecture in Japan, since it was less appealing to stakeholders than the design and construction of new buildings. This was because people were hesitant to invest in seismic evaluations to determine the vulnerability of buildings that they normally use without any problems and/or in their seismic retrofitting in case of a major earthquake that rarely occurs. However, the situation has changed since the Great Hanshin-Awaji earthquake disaster in 1995, which caused the loss of more than six thousand human lives and valuable properties. Damage surveys have shown that disasters could have been considerably mitigated if seismic evaluation and retrofitting had been carried out in advance, and the Act on Promotion of Seismic Retrofit of Buildings was enacted for existing buildings. Many buildings have been seismically evaluated, and vulnerable buildings have been retrofitted or reconstructed since then. However, it will take more time to complete this project, which is a pressing issue among earthquake-prone countries and regions. In concluding this review paper, the author would like to express that “This is one of the most important and economical measures for earthquake disaster mitigation. We have the tools. Take actions immediately”.

## Figures and Tables

**Table 1.** Basic seismic safety index (Es) for shear failure type of buildings (values in parentheses indicate Es-index for flexural failure type of buildings)^47,48)^

Predominant Period of Ground (in seconds)	0.3	0.4	0.5	0.6	0.7	0.8
1-story	0.80 (0.70)	0.70 (0.70)	0.65 (0.65)	0.60 (0.60)	0.55 (0.55)	0.50 (0.50)
2-story	0.70 (0.60)	0.70 (0.60)	0.65 (0.60)	0.60 (0.60)	0.55 (0.55)	0.50 (0.50)
3-story	0.65 (0.60)	0.65 (0.60)	0.65 (0.60)	0.60 (0.60)	0.55 (0.55)	0.50 (0.50)
4-story	0.60 (0.55)	0.60 (0.55)	0.60 (0.55)	0.60 (0.55)	0.55 (0.55)	0.50 (0.50)
5-story	0.60 (0.55)	0.60 (0.55)	0.60 (0.55)	0.60 (0.55)	0.55 (0.55)	0.50 (0.50)
6-story	0.60 (0.50)	0.60 (0.50)	0.60 (0.50)	0.60 (0.50)	0.55 (0.50)	0.50 (0.50)

**Table 2.** Construction years vs. damage grades of reinforced concrete school buildings after the 1995 Hyogo-ken Nanbu earthquake^61,62)^

	Pre-1971	1971–1981	Post-1981	Total
Collapse	18 (5%)	2 (1%)	0	20 (3%)
Severe Damage	24 (7%)	9 (5%)	0	33 (5%)
Moderate Damage	90 (27%)	39 (24%)	11 (8%)	140 (22%)
Minor Damage	41 (12%)	21 (13%)	7 (5%)	69 (11%)
Slight or No Damage	159 (48%)	95 (57%)	115 (87%)	369 (59%)

Total	332 (100%)	166 (100%)	133 (100%)	631 (100%)

## Abbreviations

JASBS: Japan Association for Special Building Safety (JBDPA since 1979)
JBDPA: Japan Building Disaster Prevention Association (www.kenchiku-bosai.or.jp)
AIJ: Architectural Institute of Japan (www.aij.or.jp)
